# Optimal Silicon Doping Layers of Quantum Barriers in the Growth Sequence Forming Soft Confinement Potential of Eight-Period In_0.2_Ga_0.8_N/GaN Quantum Wells of Blue LEDs

**DOI:** 10.1186/s11671-017-2359-3

**Published:** 2017-11-09

**Authors:** Hsiang-Chen Wang, Meng-Chu Chen, Yen-Sheng Lin, Ming-Yen Lu, Kuang-I Lin, Yung-Chen Cheng

**Affiliations:** 1 Graduate Institute of Opto-Mechatronics, National Chung Cheng University, Chiayi, 62102 Taiwan; 2 Department of Applied Science, National Taitung University, Taitung, 950 Taiwan; 3 Department of Electronic Engineering, I-Shou University, Kaohsiung, 84001 Taiwan; 4 Department of Materials Science and Engineering, National Tsing Hua University, Hsinchu, 30013 Taiwan; 5 Center for Micro/Nano Science and Technology, National Cheng Kung University, Tainan, 70101 Taiwan; 6 Department of Materials Science, National University of Tainan, Tainan, 70005 Taiwan

**Keywords:** InGaN/GaN quantum wells, Silicon doping, Blue light-emitting diodes, Soft confinement potential, Localization of carriers, Quantum-confined Stark effect

## Abstract

The features of eight-period In_0.2_Ga_0.8_N/GaN quantum wells (QWs) with silicon (Si) doping in the first two to five quantum barriers (QBs) in the growth sequence of blue light-emitting diodes (LEDs) are explored. Epilayers of QWs’ structures are grown on 20 pairs of In_0.02_Ga_0.98_N/GaN superlattice acting as strain relief layers (SRLs) on patterned sapphire substrates (PSSs) by a low-pressure metal-organic chemical vapor deposition (LP-MOCVD) system. Temperature-dependent photoluminescence (PL) spectra, current versus voltage (*I*-*V*) curves, light output power versus injection current (*L*-*I*) curves, and images of high-resolution transmission electron microscopy (HRTEM) of epilayers are measured. The consequences show that QWs with four Si-doped QBs have larger carrier localization energy (41 meV), lower turn-on (3.27 V) and breakdown (− 6.77 V) voltages, and higher output power of light of blue LEDs at higher injection current than other samples. Low barrier height of QBs in a four-Si-doped QB sample results in soft confinement potential of QWs and lower turn-on and breakdown voltages of the diode. HRTEM images give the evidence that this sample has relatively diffusive interfaces of QWs. Uniform spread of carriers among eight QWs and superior localization of carriers in each well are responsible for the enhancement of light output power, in particular, for high injection current in the four-Si-doped QB sample. The results demonstrate that four QBs of eight In_0.2_Ga_0.8_N/GaN QWs with Si doping not only reduce the quantum-confined Stark effect (QCSE) but also improve the distribution and localization of carriers in QWs for better optical performance of blue LEDs.

## Background

Silicon (Si) doping in GaN quantum barriers (QBs) can kindle Coulomb screening of polarization field and cause suppression of quantum-confined Stark effect (QCSE) in InGaN/GaN quantum wells (QWs). Radiative recombinations of excitons in InGaN/GaN QWs can be enhanced considerably through Si-doped QBs [1–4]. Compositional fluctuations of indium (In) and spinodal phase separation occur in ternary alloy InGaN on account of the inherent solid-phase miscibility gap of GaN and InN. The formation of In-rich clusters could act as strong carrier localizations, preventing the traps of nonradiative recombination centers (NRCs) and facilitating the radiative recombination of excitons in InGaN/GaN QWs [5–10]. Fluctuation of indium composition in InGaN/GaN QWs has a significant impact on the behaviors of devices, e.g., internal quantum efficiency (IQE), external quantum efficiency (EQE), and current-voltage characteristics. Indium fluctuation in InGaN/GaN QWs should be taken into account to have better explanations of the performance of devices [11]. InGaN/GaN QWs with Si-doped QBs have shown the properties of modifications of material nanostructure and formations of nanoscale islands due to the spiral growth of the QW layers [12], promotion of the thermal stability of InGaN/GaN QWs [13], improvement of light output power and electrostatic discharge (ESD) behaviors of the LED as the doping concentration in QBs is increased [14], easy blocking of hole carrier transport leading to recombination of excitons at the wells between p-type GaN (p-GaN) and the doped barriers [15], etc.

The favorable periods and thickness of InGaN/GaN QWs for high brightness and high EQE under high injection current (above several tens of mA) of blue LEDs are reported [16–18]. In the reports, nine periods of InGaN/GaN QWs grown on patterned sapphire substrates (PSSs) show a significant improvement of light emission power and droop properties of EQE [16]. The best optical and electrical performances of blue LEDs are demonstrated if the active region consists of 12 periods of InGaN/GaN QWs at the injection current 42 A/cm^2^ [17]. Apparent reduction of EQE droop and enhancement of IQE are demonstrated for the thickness of QB reduced from 24.5 to 9.1 nm in the simulation results of InGaN/GaN LEDs [18]. Si doping in proper thickness and numbers of QBs in InGaN/GaN QWs is crucial for further promotion of brightness and efficiency of InGaN blue LEDs operating at high injection current. In this report, we present the optical, electrical, and material characteristics of eight-period In_0.2_Ga_0.8_N/GaN QWs with first two to five QBs in the growth sequence possessing Si doping of blue LEDs. The results give a deeper insight into the mechanisms of carrier localization, confinement potential, and QCSE of QWs on luminescence behaviors of blue LEDs under high injection current.

## Experimental Methods

**Fig. 1:**
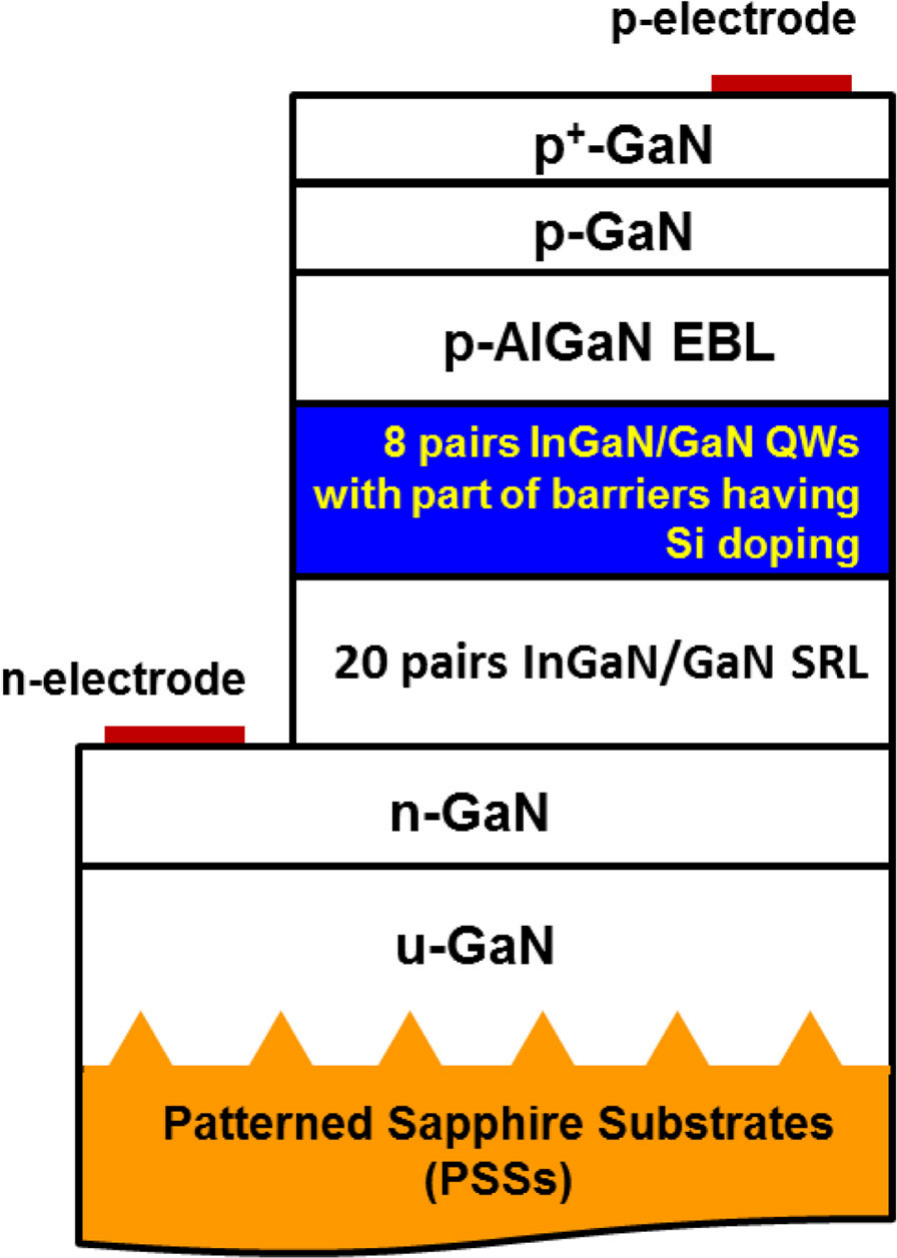
Schematic diagram of layer structures of fabricated blue LED chips. These schematic drawings of epitaxial layer thicknesses are exaggerated for clarity and are not to scale

Figure 1 depicts the schematic illustration of material layer structures of blue LED samples. Epilayers are deposited by a horizontal reactor of a low-pressure metal-organic chemical vapor deposition (LP-MOCVD) system on (0001)-orientation (c-plane) PSSs. The diameter, height, and interval of regular pyramid structures on PSSs are 2, 1.5, and 1 μm, respectively. PSSs have a thickness of 3 μm and are preheated at 1150 °C in the ambience of hydrogen before the growth of epilayers. Trimethylgallium (TMGa), trimethylindium (TMIn), trimethylaluminum (TMAl), and gaseous NH_3_ are utilized as vapor-phase precursors of elemental gallium (Ga), In, aluminum (Al), and nitrogen (N), respectively. Silane (SiH_4_) and bis-cyclopentadienyl magnesium (Cp_2_Mg) are precursors of the dopant Si and magnesium (Mg) in the n- and p-type substances, respectively. Carrier gases of precursors are the mixture of hydrogen (H_2_) and nitrogen (N_2_) with the ratio 1:1 which is chemically nonreactive.

Undoped GaN (u-GaN) buffer layer and then Si-doped n-type GaN (n-GaN) with a thickness of 3 and 3.3 μm are grown upon PSSs. The doping concentration of n-GaN is 10^19^ cm^−3^. Twenty pairs of In_0.02_Ga_0.98_N/GaN superlattice with the thickness 2/2 nm are subsequently deposited on n-GaN. It plays the role of strain relief layers (SRLs), which is used to reduce the extension of strain from the epilayers on PSSs.

Active layers of blue LEDs contain eight-period In_0.2_Ga_0.8_N/GaN QWs. The thicknesses of QW and QB are 2.5 and 8 nm with the growth temperature 750 and 900 °C, respectively. Si doping with a concentration around 3 × 10^17^ cm^−3^ in the first two, three, four, and five QBs in the growth sequence is named as samples A, B, C, and D, respectively. p-type Al_0.16_Ga_0.84_N electron blocking layer (EBL) has a thickness of 20 nm and is grown at 950 °C with Mg doping. p-GaN window layer and p^+^-GaN contact layer are grown at 950 °C with a thickness of 100 and 20 nm, and doping concentrations are 10^19^ and 10^20^ cm^−3^.

Epilayers are etched selectively to the n-GaN layer by an inductively coupled plasma (ICP) system to form mesa structure LED. The indium tin oxide (ITO) transparent contact layer (TCL) is deposited onto the p^+^-GaN surface by an electron beam evaporator. Chromium/platinum/gold (Cr/Pt/Au) multi-metal contact electrode layers are subsequently evaporated onto the p^+^-GaN and n-GaN layers for good ohmic contact. LED wafers are lapped and polished down to about 120 μm for chip dicing. Standard mesa-type LED devices with a square chip size of 1 mm^2^ are produced.

The current versus voltage (*I*-*V*) curves and the light output power (*P*_out_) versus injection current (*L*-*I*) curves from 20 to 300 mA at room temperature (RT) of diodes are measured. Temperature-dependent photoluminescence (PL) spectra of eight-period In_0.2_Ga_0.8_N/GaN QWs are examined. The excitation light source of PL is He-Cd laser (325 nm) with the average power 45 mW. The high-resolution transmission electron microscopy (HRTEM) images are taken from an EM-3000F field emission transmission electron microscope (FE-TEM) with an accelerating voltage of 300 kV and resolution of 0.14 nm.

## Results and Discussion

**Fig. 2:**
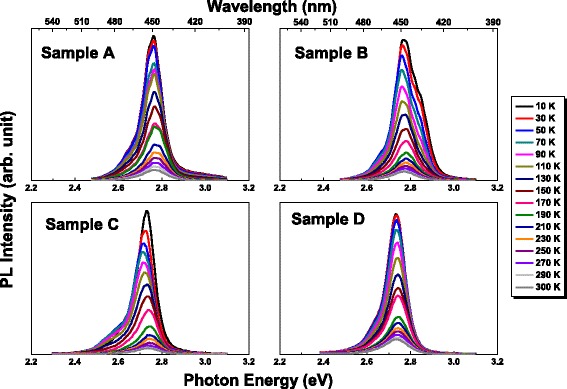
Temperature-dependent PL spectra of samples at various temperatures from 10 to 300 K

**Fig. 3:**
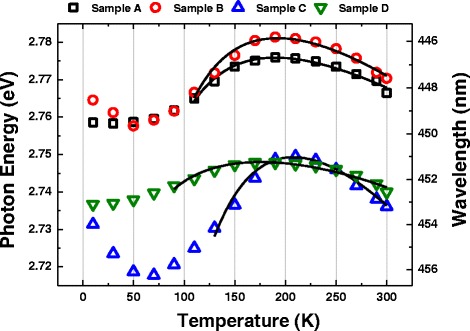
Variations of PL peak maximum energy versus temperature of samples. The best fitting curves using the Varshni equation are shown in thick and black solid lines

Figure 2 plots the PL spectra of samples at various temperatures from 10 to 300 K. The monotonic decrease of PL peak intensity with the increase of temperature can be observed. This is originated from the raise of nonradiative recombination process with the increase of temperature. All PL spectral profiles are fitted by Gaussian lineshape functions to find the variations of peak maximum energy with temperature as demonstrated in Fig. 3. The emission peak maximum energy of PL for the undoped In_0.2_Ga_0.8_N/GaN QWs is 2.68 eV at room temperature. This means that PL peak energies of samples with first two to five QBs having Si doping exhibit a blue shift when it is compared with the undoped one. The first two to five QBs with Si doping can lower the QCSE in QWs effectively. It should be pointed out that the uses of PSSs and SRLs in the structures of epilayers can reduce the part of piezoelectric (PZ) field as well as QCSE in In_0.2_Ga_0.8_N/GaN QWs.

In Fig. 3, variations of PL peak maximum energy versus temperature that exhibit S-shape curves of all samples can be shown. The S-shape curves display initial red then blue and then red shift with the increase of temperature. This behavior is attributed to temperature-dependent collective luminescence of strong localized excitons in QWs. The Varshni empirical equation shown below can be used to fit the range of blue to red shift of the S-shape variations [19–21].

1 $$ {E}_{\mathrm{g}}(T)={E}_{\mathrm{g}}(0)-\frac{\alpha {T}^2}{\left(T-\beta \right)}-\frac{\sigma^2}{k_{\mathrm{B}}T} $$

where *E*_g_(0), *α*, *β*, and *σ* are the fitting parameters. *E*_g_(0) is the band gap energy of QW at the absolute temperature 0 K. *α* and *β* are the thermal coefficients which are material-dependent parameters. *σ* is the Gaussian broadening parameter, and it physically reflects the degree of thermal distribution of carriers within the band tail-localized states. The second term in Eq. (1) indicates that the band gap energy of semiconductors tends to decrease with increasing temperature and it is known as the energy gap shrinkage on account of the raise of the amplitude of atomic vibrations. The third term is used to characterize the degree of carrier localization with temperature under the assumption of nondegenerate carrier distribution and Gaussian-like localized density of state. Strong carrier localization is a crucial aspect in realizing the raise of radiative recombination and IQE in InGaN QWs due to the improvement of the overlap between electron and hole wave functions. The Varshni equation is not applicable in the range of red shift of PL peak energy at low temperature, owing to the strong degeneracy in carrier distribution. The best fitting results of samples are illustrated in thick and black solid curves in Fig. 3. The localization energy of samples A, B, C, and D is 24, 28, 41, and 13 meV, respectively. Sample C possesses the largest localization energy, *σ* (41 meV). Carrier localization in the QWs with four Si-doped QBs is stronger than the others. The smallest localization energy (*σ*) happens in sample D with the value 13 meV.

**Fig. 4:**
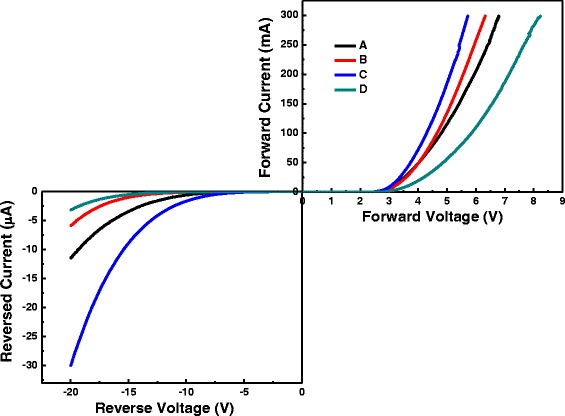
Current-voltage (*I*-*V*) curves under forward and reversed biases of diodes

Current-voltage (*I*-*V*) characteristics under forward and reverse bias of blue LEDs are demonstrated in Fig. 4. In this figure, the turn-on voltages for samples A, B, C, and D are 3.41, 3.47, 3.27, and 4.03 V at the forward current 20 mA, respectively. The breakdown voltages for samples A, B, C, and D are − 8.85, − 9.99, − 6.77, and − 11.55 V at the reverse current 1 μA, respectively. Sample C has the smallest turn-on and breakdown voltages. Sample D has the largest turn-on and breakdown voltages. Accordingly, lower barrier height of QBs is suggested in sample C. Soft (smooth) confining potential of QWs is expected in the sample with the first four QBs having Si doping. Soft confinement potential profile implies that conduction and valence band offsets of QWs are not taken as sharp step functions, i.e., not rectangular confining potential. Smooth confining potentials can suppress the Auger recombination to a great extent and prevent accumulation of plenty carriers in the first several wells in the injection direction of InGaN/GaN QWs [22–24]. Smooth confining potential can also be made through a linear decrease of In composition along the growth direction of InGaN/GaN QWs. This can have better spread of carriers among QWs and transportation of holes which results in improvement of quick drop of EQE and light output power [25–30].

**Fig. 5:**
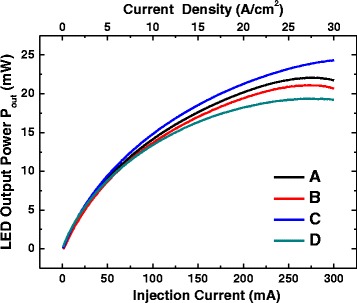
Light output power (*P*_out_) versus injection current (*L*-*I*) curves from 0 to 300 mA of diodes

Figure 5 shows the measurements of light output power (*P*_out_) of LEDs versus injection current (*L*-*I*) curve from 0 to 300 mA by a chip tester. The increase of the *P*_out_ with the raise of injection current of *L*-*I* curves can be observed. The highest and the lowest *P*_out_ for high injection current are shown in samples C and D, respectively. Saturations of *P*_out_ at the injection current 300 mA are shown in all samples except for sample C. Saturation effect of *P*_out_ is referred to the current overflowing the QWs. The less current overflowing the QWs takes place in the soft confinement potential of QWs and strong carrier localization inside the QW sample. Better spread of carriers among eight QWs and strong radiative recombination of carriers inside QWs are displayed in the sample with four QBs containing Si doping.

**Fig. 6:**
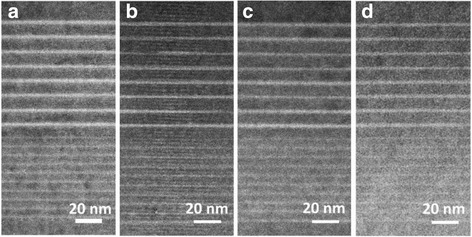
Dark field images of the samples. **a** Sample A. **b** Sample B. **c** Sample C. **d** Sample D

**Fig. 7:**
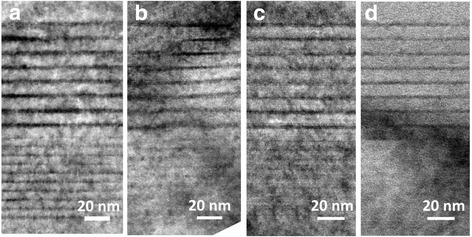
Bright field images of the samples. **a** Sample A. **b** Sample B. **c** Sample C. **d** Sample D

**Fig. 8:**
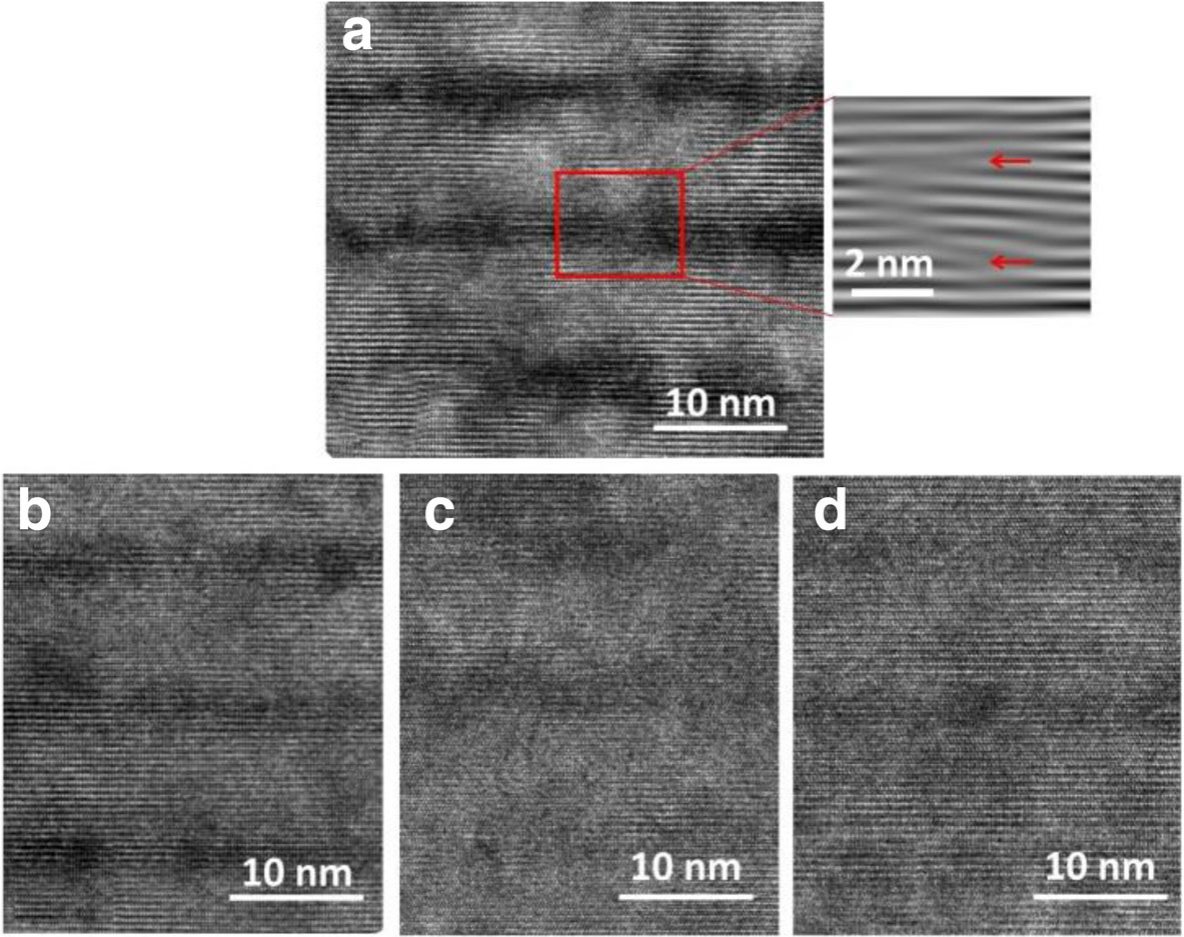
Enlarged bright-field images of the samples. **a** Sample A. **b** Sample B. **c** Sample C. **d** Sample D

The dark-field, bright-field, and enlarged bright-field images of cross-sectional high-resolution transmission electron microscope (HRTEM) of samples are shown in Figs. 6, 7, and 8. In Fig. 6, bright and dark contrasts correspond to InGaN and GaN epilayers. Eight periods of QWs and 20 pairs of superlattices can be observed. In Fig. 7, dark contrast variations represent the fluctuations of local In content and formation of indium-rich clusters in QWs [31–34]. Indium-rich clusters act as relatively deep localized states of carriers leading to high radiative excitonic recombination. Lattice distortion and stacking fault defects are noted around these indium-rich clusters, indicating that the strain energy partly relaxed in the InGaN layer as shown in the zoom in picture of the red square in Fig. 8a. Compared with the enlarged bright-field images, more diffusive (not abrupt) interfaces of QWs in samples C and D are seen as exhibited in Fig. 8c, d. The agent comes from the well-known interdiffusion of In and Ga atoms at the well/barrier interface. Very weak confinement and carrier localization of QWs occurred in sample D which is in accordance with the worst optoelectronic behavior of this sample. In the images, threading dislocations (TDs) do not form in all samples. NRCs occur primarily at TD sites. Better quality of epilayers is shown in these samples as expected due to the employing of PSSs and SRLs [35–37]. Four QBs with Si doping in eight-period QWs are the favorable condition to modify the confinement potential to attain soft confinement potential of InGaN/GaN QWs, leading to the best optoelectronic performance of blue LED.

## Conclusions

Temperature-dependent PL spectra, *I*-*V* curves, *L*-*I* curves, and HRTEM images of blue LEDs with eight-period In_0.2_Ga_0.8_N/GaN QWs having the first two to five QBs in the deposition sequence containing Si doping were investigated. The results demonstrate that the sample with the first four QBs containing Si doping has relatively lower barrier height and larger localization energy of carriers which is evidenced by diffusive interfaces of QWs in HRTEM images. Soft confinement potential of QWs accompanied with stronger carrier localization inside QWs gives rise to a more uniform distribution of carrier among eight QWs and higher radiative recombination of carriers inside QWs in this sample. A decrease of Auger processes and leakage of carriers as well as an increase of radiative recombination of QWs occurred in blue LEDs with four Si-doped QBs under high injection current. The results provide optimal conditions of QBs with Si doping through the analyses of the effects of carrier localization, confinement potential, PZ field, and material nanostructures on the luminescence properties of In_0.2_Ga_0.8_N/GaN QWs of blue LEDs.

## Abbreviations

Al: Aluminum
Cp_2_Mg: Bis-cyclopentadienyl magnesium
EBL: Electron blocking layer
EQE: External quantum efficiency
FE-TEM: Field emission transmission electron microscope
Ga: Gallium
HRTEM: High-resolution transmission electron microscopy
ICP: Inductively coupled plasma
In: Indium
IQE: Internal quantum efficiency
ITO: Indium tin oxide
*I*-*V*: Current versus voltage
*L*-*I*: Light output power versus injection current
LP-MOCVD: Low-pressure metal-organic chemical vapor deposition
Mg: Magnesium
N: Nitrogen
n-GaN: n-type GaN
NRCs: Nonradiative recombination centers
PL: Photoluminescence
PSSs: Patterned sapphire substrates
PZ: Piezoelectric
QBs: Quantum barriers
QCSE: Quantum-confined Stark effect
QWs: Quantum wells
RT: Room temperature
Si: Silicon
SiH_4_: Silane
SRLs: Strain relief layers
TCL: Transparent contact layer
TMAl: Trimethylaluminum
TMGa: Trimethylgallium
TMIn: Trimethylindium
u-GaN: Undoped GaN
